# Optimization of patient-specific stereo-EEG recording sensitivity

**DOI:** 10.1093/braincomms/fcad304

**Published:** 2023-11-02

**Authors:** Grace E Dessert, Brandon J Thio, Warren M Grill

**Affiliations:** Department of Biomedical Engineering, Duke University, Durham, NC 27708, USA; Department of Biomedical Engineering, Duke University, Durham, NC 27708, USA; Department of Biomedical Engineering, Duke University, Durham, NC 27708, USA; Department of Electrical and Computer Engineering, Duke University, Durham, NC 27708, USA; Department of Neurobiology, Duke University, Durham, NC 27710, USA; Department of Neurosurgery, Duke University, Durham, NC 27710, USA

**Keywords:** stereo-electroencephalography (sEEG), computational modelling, interictal spike, dipoles

## Abstract

Stereo-EEG is a minimally invasive technique used to localize the origin of epileptic activity (the epileptogenic zone) in patients with drug-resistant epilepsy. However, current stereo-EEG trajectory planning methods are agnostic to the spatial recording sensitivity of implanted electrodes. In this study, we used image-based patient-specific computational models to design optimized stereo-EEG electrode configurations. Patient-specific optimized electrode configurations exhibited substantially higher recording sensitivity than clinically implanted configurations, and this may lead to a more accurate delineation of the epileptogenic zone. The optimized configurations also achieved equally good or better recording sensitivity with fewer electrodes compared with clinically implanted configurations, and this may reduce the risk for complications, including intracranial haemorrhage. This approach improves localization of the epileptogenic zone by transforming the clinical use of stereo-EEG from a discrete *ad hoc* sampling to an intelligent mapping of the regions of interest.

## Teaser

Dessert *et al*. provide the first method for optimized stereo-EEG planning that considers spatial recording sensitivity based on patient-specific biophysical modelling. Optimized electrode configurations may improve both the safety and the efficacy of epileptogenic zone localization compared with manual planning.

## Graphical Abstract

**Graphical Abstract:**
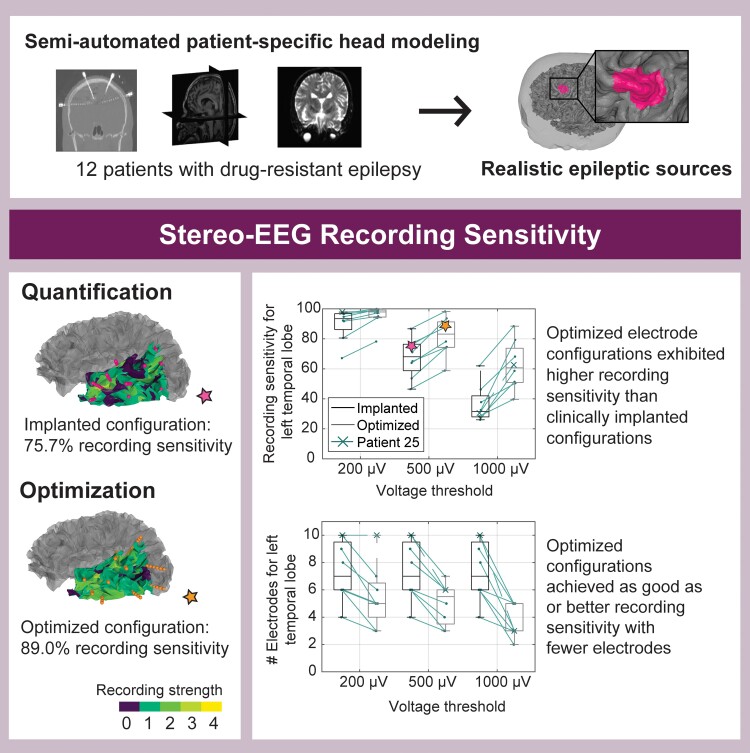

## Introduction

More than 30% of the 65 million people with epilepsy worldwide do not benefit from pharmaceuticals.^1-3^ Surgical resection/ablation is the primary curative treatment for pharmaco-resistant epilepsy but requires robust localization of the epileptogenic zone (EZ)—the minimum amount of neural tissue that needs to be removed to achieve seizure freedom.^1,3^ Non-invasive EZ localization is often insufficient and invasive monitoring is often required.^2^ Stereo-EEG (sEEG) is an EZ localization approach by which up to 30 electrodes are implanted to record electrical activity from widespread areas of the brain, and sEEG has largely displaced other forms of invasive monitoring because of reduced complication rates.^4^ Implanting only the essential number of sEEG electrodes is critical because each additional electrode increases the risk of haemorrhage, while too few electrodes can lead to poor localization.^3-5^

In this study, we developed a novel approach to optimize the recording sensitivity (RS) of sEEG electrode configurations and thereby minimize the number of implanted electrodes while maintaining recording coverage and accurate localization. Automated planning algorithms for determining sEEG electrode implantation trajectories given a user-specified region of interest (ROI)^6-9^ have shown promise to increase grey-matter sampling, decrease risk scores and reduce planning time compared with manual planning.^10^ However, these algorithms did not account for the spatial extent of tissue that can be recorded (RS), which is critical to EZ localization.

We implemented patient-specific head models to simulate the spatial distribution of voltages generated by spatially extended sources of epileptiform neural activity. We quantified the RS of arbitrary sEEG configurations and developed an optimization method to identify electrode trajectories that maximize the RS of user-defined ROIs while avoiding critical anatomy. This approach transforms sEEG planning from a manual *ad hoc* process to an intelligent mapping to improve EZ localization and patient safety.

## Materials and methods

### Semi-automated patient-specific volume conductor head modelling

**Figure 1:**
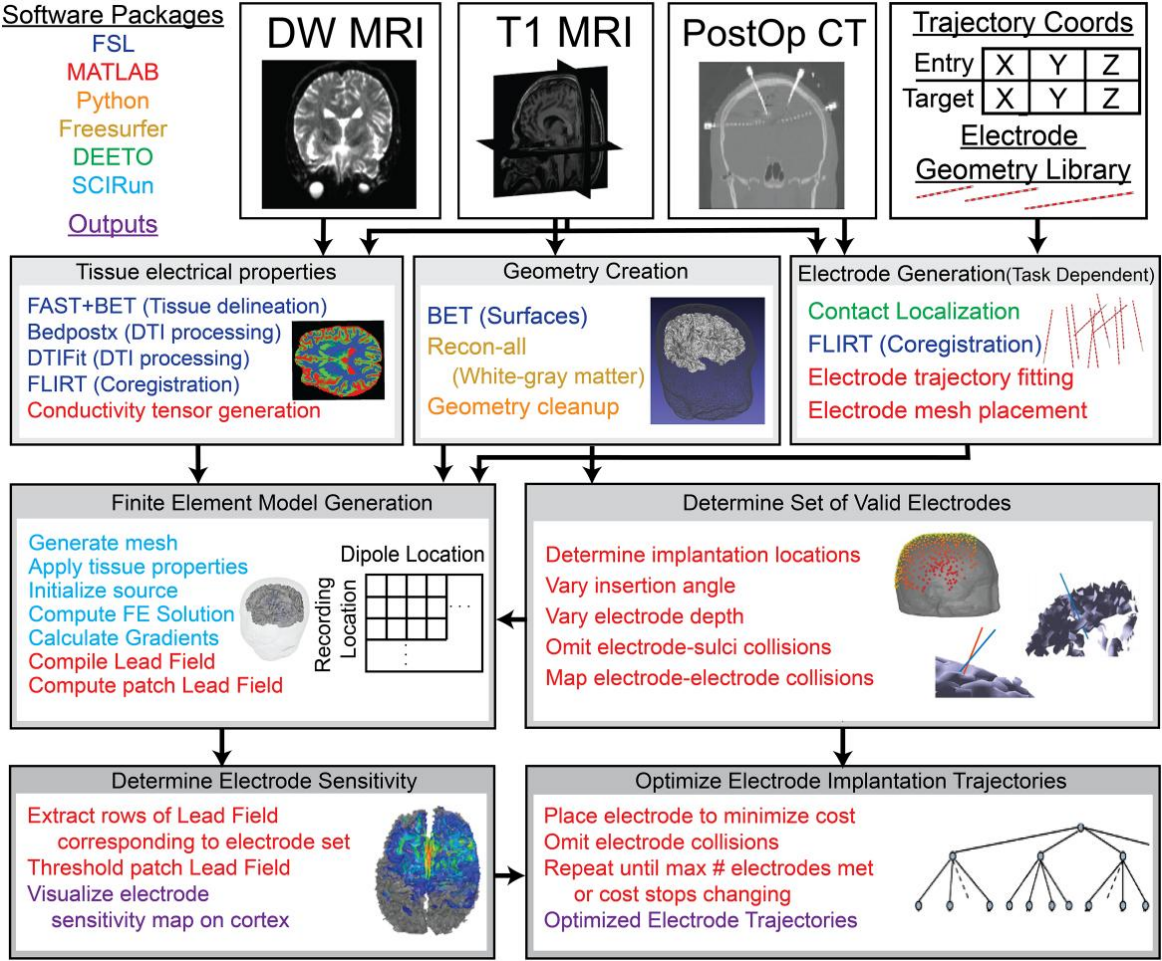
**Pipeline for optimization of sEEG electrode implantation.** We used T_1_ MRI, PostOp CT and DW-MRI imaging to generate 12 patient-specific FEM head models. We generated a set of valid electrode trajectories, computed RS and optimized configurations for specific ROIs.

The Duke University Health System IRB approved the use of clinical neuroimaging in this study to do secondary research on data collected as part of research study Pro00101171, and the participants whose neuroimaging was used provided written informed consent. From these data, we selected the 12 epilepsy patients over the age of 18 who had full sets of neuroimaging. We developed a semi-automated pipeline to implement patient-specific head models, combining patient-specific neuroimaging [T_1_ MRI, diffusion-weighted MRI (DW-MRI) and PostOp CT], implantation planning coordinates and custom code (Fig. 1). Patient-specific head modelling was divided into four modules: (i) geometry creation, (ii) defining the tissue electrical properties, (iii) electrode generation and (iv) finite-element model (FEM) generation.

We created the model geometry using the T_1_ MRI. We extracted the skin surface using FSL (BET) and defined the white–grey matter boundary using freesurfer’s (https://surfer.nmr.mgh.harvard.edu/) recon-all function. The white–grey matter boundary mesh was manually smoothed by decimating and up-sampling the mesh in MeshLab (https://www.meshlab.net/).

We defined the tissue electrical properties by segmenting the T_1_ MRI into five different tissue types (skin, skull, CSF, white matter and grey matter) using FSL’s (https://fsl.fmrib.ox.ac.uk) analysis tools (FAST and BET). We then obtained diffusion tensors from the DW-MRI using FSL and coregistered the tensors in T_1_ space. Finally, we converted the diffusion tensors to conductivity tensors using the load preservation technique to define anisotropic conductivity tensors for each patient-specific model.^11^

We generated electrode geometries using the PostOp CT and a library of predefined electrode geometries created by PMT and ADTech. We used DEETO^12^ to localize the sEEG electrode contacts given the entry and target locations of each electrode along with the PostOp CT. We then co-registered the electrode contact locations in T_1_ space and fitted a line to each electrode. If we were simulating the clinically implanted electrodes, we could then place a mesh electrode geometry on each trajectory. For arbitrary trajectory simulations, we chose not to include the electrode geometries in the FEM, because changing the geometry for each simulation would require us to remesh each simulation. Additionally, previous work has shown that electrodes have minimal influence on recorded potentials at distances >5 mm.^13,14^

Finally, we combined all the processed components into a FEM in SCIRun v5.0 (SCI Institute, University of Utah, Salt Lake City, UT, USA) with 19–22 million elements. We used SCIRun to solve for the voltages throughout the head model generated by dipole sources pointed orthogonally outward from the cortical surface at ∼40 000 cortical locations. We grounded points at the base of the skin mesh to serve as a voltage reference in the FEM simulations. We compiled the simulated voltages for each source as columns into a lead-field matrix, which defined the input–output relationship between neural sources and recorded voltages.

### Extended source modelling

Simplified source models are required to simulate the voltage distribution generated by realistic epileptic sources. Dipoles are appropriate source representations of active neurons for sEEG, and extended dipole models are necessary to model patches of active cortex when recording within 1.5 cm.^15^ We defined three extended dipole models, or patch models, centred at every element on the cortical triangular surface mesh. These were sets of adjacent dipoles on the cortical surface with surface areas of 6, 10 and 20 cm^2^, which correspond to areas projected on the inside of the skull of 2.46, 3.85 and 7.09 cm^2^ (Supplementary Fig. 5). We also quantified the spatial extents of each patch using the mean distances between the centre of the mass and the edges of each patch.

We calculated the voltages generated by each patch model by finding the columns of the lead-field matrix corresponding to each dipole within a patch, and we scaled each column by the area of the corresponding triangle of the cortical mesh. Then, we summed the columns and scaled them by an estimate of human neocortex dipole moment density (0.16–0.77 nA-m/mm^2^) to obtain the voltage.^16^ By concatenating the calculated distributions of voltages for all extended dipole models as columns, we assembled the patch lead-field matrix for all pairs of three patch areas and three dipole moment density values. We used the minimum, mean and maximum values of 0.16, 0.465 and 0.77 nA-m/mm^2^ to capture the range of dipole moment density values. We used the mean values of 10 cm^2^ patch area and 0.465 nA-m/mm^2^ dipole moment density in all analyses to capture the best-estimated results.

### Estimation of the recording radius

We estimated the radius around a contact within which discernible neural signals could be recorded. We defined discernible voltage as the amplitude of a spike that would consistently be discriminated from background noise in sEEG signals. Current clinical practice relies on the identification of large interictal spikes typically >1 mV amplitude (Supplementary Fig. 1). We analysed interictal sEEG voltage data from 12 patients to determine the distribution of noise and spike amplitude. We determined the amplitude of the noise by considering the standard deviation of the recorded voltages when there was no spiking activity and multiplied it by 4 (Supplementary Fig. 1A). The noise distribution was roughly Gaussian, and therefore, four times the standard deviation will account for 99.99% of the data. Based on the standard deviation of the noise, interictal spikes >200 µV in magnitude (±4 SDs) can be differentiated from noise (Supplementary Fig. 1B). Using 120 clinician-defined spikes across 10 patients, we calculated the maximum voltage across all the sEEG recordings using a common average referencing scheme. The 25 percentile maximum voltage was 548 µV, and the mean maximum voltage was 958 µV. Therefore, we used voltage thresholds of 200, 500 and 1000 µV in all analyses.

To estimate the recording radius, we first selected 105 simulated contact locations from each hemisphere of each patient using 3 random points on the cortex surface from each of 35 cortical subregions based on the Desikan–Killiany atlas.^17^ We selected the rows from the patch lead-field matrices corresponding to these locations and applied one of the three discernible voltage thresholds to determine the patches that were recordable by each contact location. We calculated the distance from the centre element of every patch to each contact location and used this to sort the patches into groups of radii from each contact with a 0.25 cm bin size. For each contact, there was at least one patch in every group of radii, and each patch was a member of only one group. We calculated the per cent of patches within each group that were recordable by each contact (RS). We repeated this procedure for the same contact locations using all nine source types and all three discernible voltage thresholds and calculated the median RS across radii. We determined the recording radius as the maximum distance at which ≥50% of sources (median) had ≥20% RS.

### Quantification of configuration RS

We developed a visualization tool and metric to quantify the RS or the extent of tissue in a ROI that is recordable by a given electrode configuration. sEEG source localization requires multiple contacts to record discernible signals from a source, and therefore, we defined the recordable patches for an electrode configuration to be those that generated discernible signals at a minimum of two contacts on any single electrode. To find the set of recordable patches, we selected all columns of the patch lead-field matrix corresponding to sources with central elements inside the ROI and applied a discernible recording threshold (200, 500 or 1000 µV). Then, for each electrode, we summed the rows corresponding to its 16 contacts, concatenated them and applied a threshold of 2 to obtain a logical matrix representing the recordable patches for every electrode.

An element of the cortex was recordable by a configuration if the patch model centred at that element was recordable, and the recording strength of the patch was the number of electrodes for which that patch was recordable. We visualized the RS of an electrode configuration by plotting the recording strength of each element on the cortex on a 3D surface plot. We quantified RS*_i_*_,*thr*_(ROI) as the percentage of patches in a ROI that were recordable by a configuration:


(1) $$RS_{i,thr}(\mathrm{ROI})=P_{i,thr}(\mathrm{ROI})/P_{\mathrm{total}}(\mathrm{ROI})$$

where *P_i,thr_*(ROI) is the number of patch models in a ROI that is recordable by at least two contacts on any electrode of a configuration, *i*, at a certain threshold, *thr*, and *P*_total_(ROI) is the total number of patch models with centre elements inside the ROI. *P*_total_(ROI) is also equal to the total number of elements inside the ROI because we generated one patch around each element.

### Optimization of electrode number and placement

We generated sets of valid electrode placements and built optimized electrode configurations for 12 patients who were given clinician-defined ROIs.

### Generation of a valid electrode set

To finely sample the search space of a valid implantation area and at the same time keep the lead-field matrix <250 GB, we computed a set of 95 000 valid electrode trajectories per patient. Valid electrodes satisfied the safety criteria of insertion location, maximum insertion angle, maximum trajectory length and maximum distance to critical structures. We modelled electrodes with 16 contacts each, of 2 mm contact length and of 1.5 mm insulator length based on PMT-2102-16-091 electrodes.^18^ We simplified each contact to a single-point recording location at its centre, an approximation that was previously validated.^14^ We defined valid entry locations by selecting all points on the patient scalp surface that were within 5 mm of a best-fit standard template of the implantation scalp area.^7,19^ We randomly sampled 800 insertion locations from this region and 366 insertion angles for each to evenly sample the implantation space with ∼300 000 electrode lines (defined by insertion location and angle), assuming that most would be excluded. We kept the implantation angle ≤10° from normal to the scalp.^8^ We discretized these lines into individual electrode trajectories by sampling insertion depth at multiples of 3.5 mm, keeping the total trajectory length <10 cm.^20,21^ Because the contact points were also 3.5 mm apart, most contact points for electrodes in the same line overlapped, allowing us to represent a greater number of electrodes with fewer recording locations.

We eliminated electrodes that intersected critical structures—sulci, the midline and secondary skull locations. Angiogram imaging of blood vessels was not available, and because sulci are often used to estimate the locations of large vasculature and as critical structures themselves,^6,7,9^ we calculated the occurrence of intersections between electrodes and sulci surfaces to avoid areas where large blood vessels are likely to be located. We generated the sulci surfaces for each hemisphere individually by considering the intersection of the cortex surface with a super-smoothed cortex surface (MeshLab, filter ‘hc_laplacian_smoothing’ 100 times). The resulting surfaces were the bases of sulci. To calculate the intersections, we used a bounding volume hierarchy method adapted from Sparks *et al*.,^7^ in which the minimum distance from the electrode to the surface was found using an efficient binary tree search.^7^ We eliminated all electrodes that passed within 1.5 mm of the sulci surface^6^ or 4 mm of the skull (away from insertion location) or the midline. We then randomly eliminated lines until we were left with 95 000 valid electrode trajectories. Lastly, we calculated the occurrences of collisions (distance <4 mm)^6^ between every pair of electrodes by representing each electrode as a set of 128 points^7^ and performing matrix distance calculations.

### Optimization problem and cost function

We designed a search algorithm to find the best configurations for three ROIs per patient [left temporal lobe (LTL), clinician-defined ROI and left hemisphere (LH)] given the sets of all valid electrode trajectories and occurrences of collisions between every pair. To quantify how well a certain configuration, *i*, can record from a ROI with threshold, *thr*, we used the following cost function, *C_i_*_,*thr*_(ROI), to represent the number of patches with centre elements in the ROI that are not recordable:


(2) $$C_{i,thr}(\mathrm{ROI})=P_{\mathrm{total}}(\mathrm{ROI})-P_{i,thr}(\mathrm{ROI})$$

where *P_i_*_,*thr*_(ROI) and *P*_total_(ROI) are as defined in Eq. (1).

We conceptualized the generation of an electrode configuration as a tree traversal through an ‘*N*-ary’ tree, where each node represents a valid trajectory and each node has *N*-*x* children (i.e. the number of valid electrodes minus some number ‘*x*’ that intersect with electrodes in the current path). Each edge represents the addition of an electrode to a configuration, and each root-to-leaf path represents a full configuration where either there are no other valid electrode choices that would reduce the cost function or the full ROI is recordable. The optimal configuration is the path with the lowest cost at a certain number of electrodes (level of tree).

### Next-best search algorithm

For any configuration of three or more electrodes, it is computationally intractable to find the true best configuration because the number of paths grows exponentially with the number of electrodes. Thus, we conducted a next-best iterative tree search to find solution configurations. A single optimization trial required the selection of many parameters—source area, source strength, ROI and a threshold-priority order, which assigns the three thresholds (200, 500 and 1000 µV) primary, secondary and tertiary importance. At each level of the search tree, we picked the electrode to decrease the cost function maximally at the priority threshold. We broke ties between electrodes by using the cost functions at the second and then third priority thresholds and finally a random choice, if necessary. The next-best search algorithm is not guaranteed to produce the optimal electrode configuration because the mapping problem does not have an optimal substructure. However, given that finding the true best configuration is computationally intractable, this approach identifies a good option. We conducted the next-best search for each patient’s LTL, LH and clinician-defined ROI using all six permutations of cost functions (all permutations of 200, 500 and 1000 µV for priority order) and all nine source types (all choices of three patch area and three dipole moment density parameters). We continued adding electrodes until we added 31 electrodes or until no remaining electrodes could improve the cost function. Because our algorithm was iterative, the optimized configuration of any number of electrodes, *X* between 1 and 31, was the set of the first *X* electrodes in the configuration. We manually defined the LTL surfaces based on cortex geometry. We manually defined the ROI surfaces by selecting targeted areas of the cortex defined in clinician notes (Supplementary Table 1) and excluding subregions that did not contain electrode trajectories. We analysed the optimized configurations, using the minimum cost function for each threshold of interest.

### Quantification of clinically implanted configuration RS

We compared the recording sensitivities [Eq. (1)] of our optimized configurations to those of the clinically implanted sets. We defined the order of implanted electrodes for a certain case by sorting them in the best possible order to maximally decrease the cost function (increase the RS) with each addition.

Eight patients had clinician-defined ROIs that included, but were not limited to, one of the temporal lobes (TLs). For these patients, we defined two versions of the implanted configuration, one including only those electrodes (4–10) inside the TL ROI and the other including all electrodes (12–15) in a broad ROI. The broad ROIs for three of these patients included secondary areas on the other hemisphere, and we excluded these areas and the corresponding electrodes from consideration. We included an electrode in the TL configuration if at least one contact was within 3 mm of the ROI surface and at least half of the contacts were within 10 cm of the ROI. We compared the RS between the optimized configurations for the TL ROIs and the broad clinician-defined ROIs to recording sensitivities of the implanted configurations. The remaining four patients had ROIs outside of or not focused on a TL. For these, we computed the RS of the full implanted configurations of 11–16 electrodes for the clinician-defined ROI.

### Transfer of patient-specific configurations to other patients

To determine the importance of patient-specific configuration generation, we transferred the optimized configurations for the LH and LTL ROIs for each patient to all other patients. We used FSL’s FLIRT function with six degrees of freedom and a mutual information cost function to map configurations to other patients. Limiting the transformations to simple rotation and translation ensured consistent electrode geometries. We used 10 cm^2^ patch area, 0.465 nA-m/mm^2^ dipole moment density and a 500 µV priority cost function to generate optimized configurations. After transferring each configuration to all other patients, we found the set of all contact locations for the optimized and transferred configurations and recomputed the lead-field matrix at these locations. We calculated the per cent RS for the LTL and LH configurations in all patients and found the per cent error between the optimized and the transferred cases for each configuration. For each configuration, we compared RS values at the minimum number of electrodes that produced ≥75% RS of the ROI in the matched-patient case. We also tested the validity of all transferred configurations by calculating the minimum distance of electrodes from patient sulci surfaces. Any configuration with an electrode that passed within 2.5 mm of a sulci surface was considered invalid, but we included all electrodes for analysis to understand the best-case RS.

### Statistical analysis

**Figure 2:**
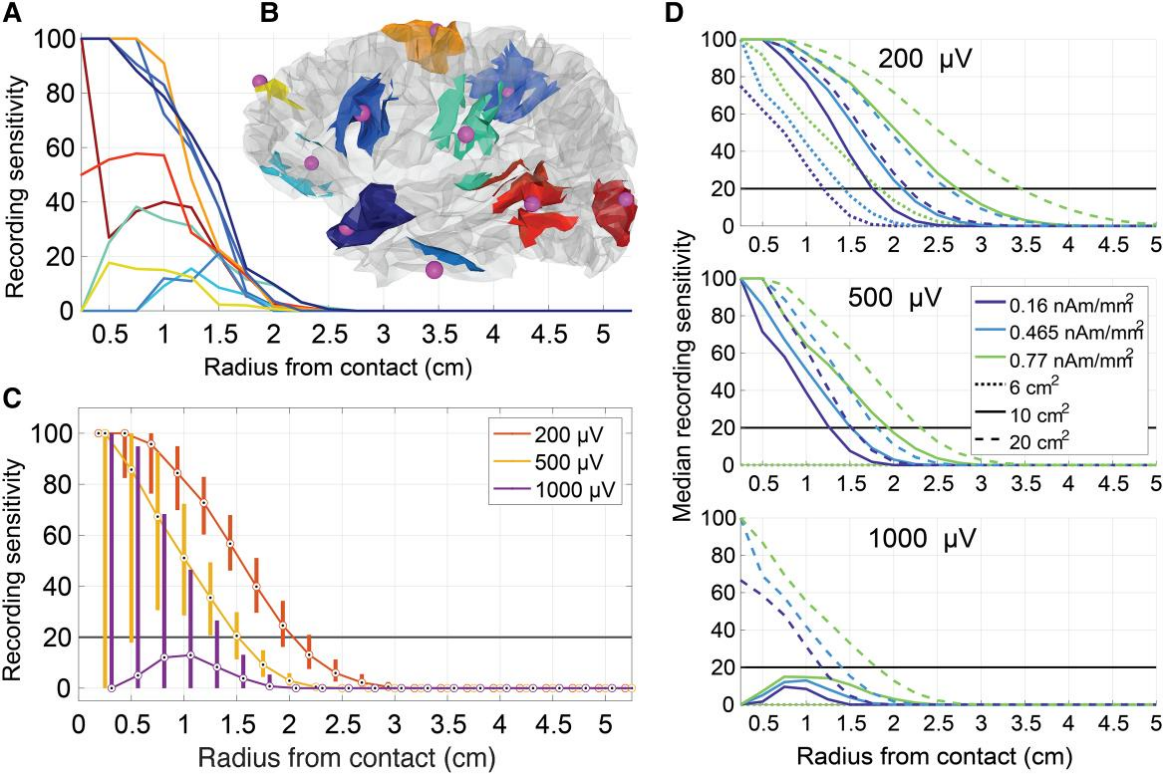
**RS of sEEG electrode contacts.** (**A**) RS as a function of distance between the centre of the simulated epileptiform activity and the electrode contact for 10 representative contacts using a 500 µV signal detection threshold. (**B**) Area of RS (cortical patches) for 10 contacts (dots) corresponding to each trace seen in **A** using a 500 µV signal detection threshold. (**C**) Median and interquartile range of RS as a function of source-to-contact distance for 105 contacts in each of 12 patients (*n* = 1260). (**D**) Median RS as a function of source to contact distance for a range of source modelling parameters and signal detection thresholds for 105 contacts in each of 12 patients (*n* = 1260). The source modelling parameters are 10 cm^2^ and 0.465 nA-m/mm^2^ for **A–C**. The 20% RS threshold is highlighted as a horizontal line for **C** and **D**.

**Figure 3:**
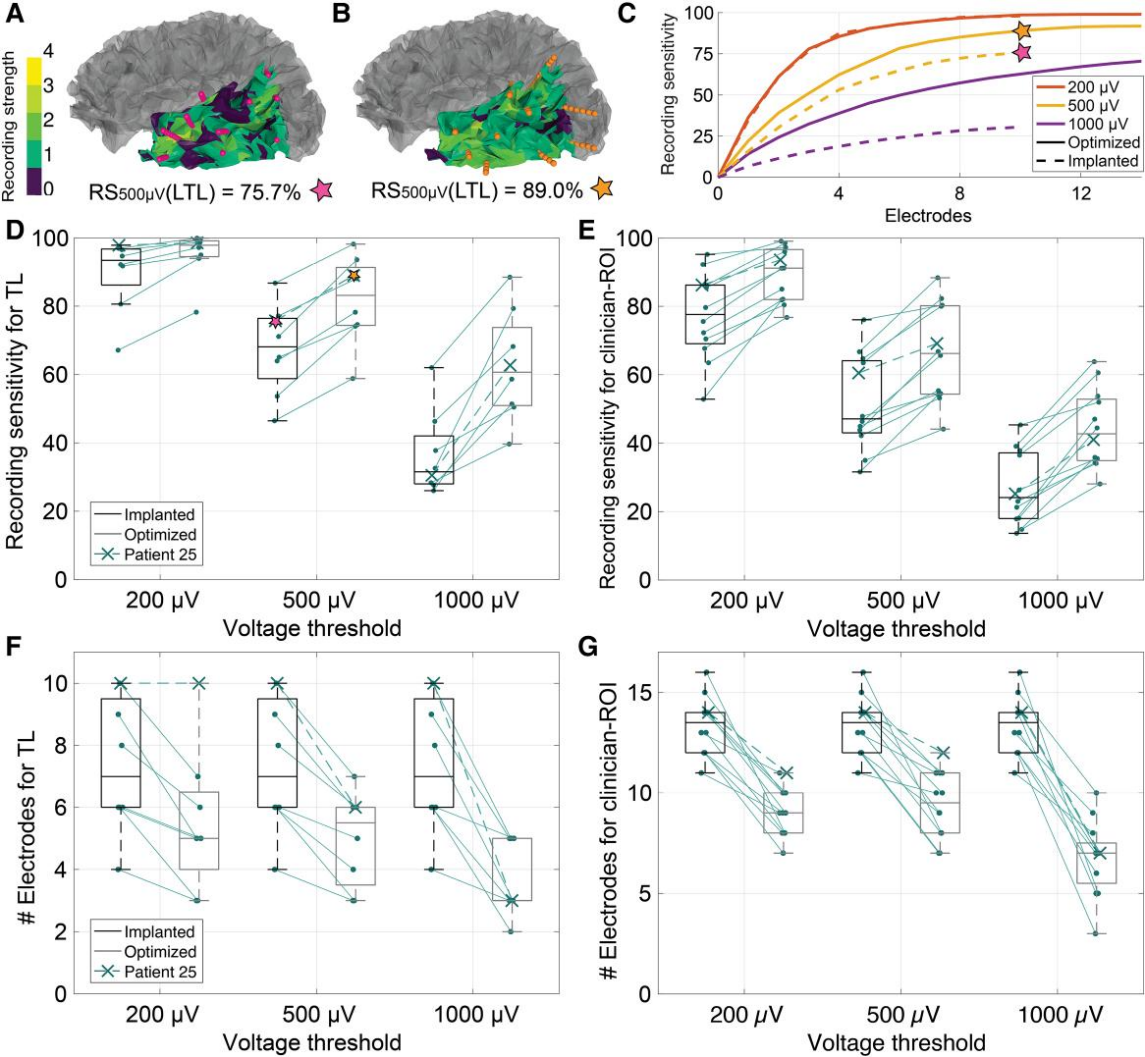
**RS of optimized and clinically implanted electrode configurations.** (**A** and **B**) Recording strength (number of electrodes that can record each source) across Patient 25 LTL with RS [Eq. (1)] for clinically implanted (**A**) and optimized (**B**) TL configurations with 10 electrodes at 500 µV threshold. Coloured cortex indicates recording strength in the ROI, grey area is outside the ROI, and contact points are shown as spheres. (**C**) RS as a function of number of implanted electrodes for Patient 25 LTL with the optimized (solid) and implanted (dashed) configurations at multiple voltage thresholds. Stars correspond to RS_500 µV_ of the configurations in **A** and **B**. (**D** and **E**) RS at three thresholds for optimized (black) and clinically implanted (grey) electrode configurations using the same number of electrodes. All differences were significant (*P* < 0.02; two-tailed paired *t*-test for Gaussian samples and Wilcoxon signed rank tests for non-Gaussian samples). (**D**) TL-specific configurations (*n* = 8). (**E**) Configurations for full clinician-defined ROIs (*n* = 12). (**F** and **G**) Number of electrodes for clinically implanted (grey) and optimized (black) configurations with equivalent or improved RS at three voltage thresholds. All differences were significant (*P* < 0.02; two-tailed paired *t*-test for Gaussian samples and Wilcoxon signed rank tests for non-Gaussian samples). (**F**) TL-specific configurations (*n* = 8). (**G**) Configurations for full clinician-defined ROIs (*n* = 12). The box plots in all figures indicate the median, interquartile range, maximum and minimum values, with outside points indicating outliers. The individual tests used, *P*-values and test statistics are available in Supplementary Table 3.

**Figure 4:**
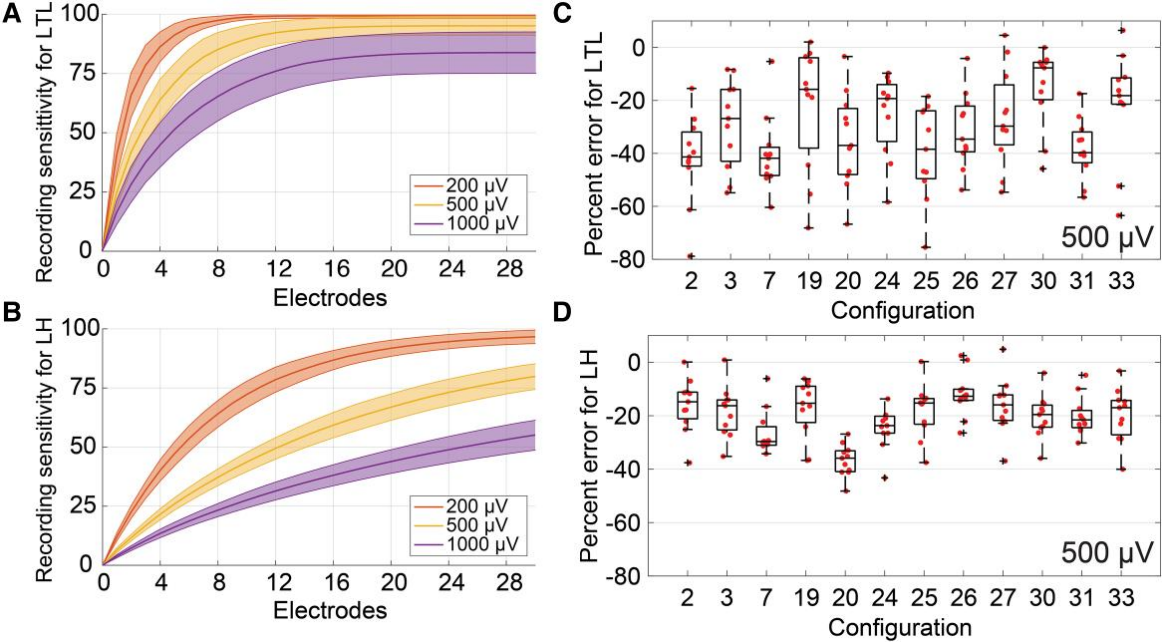
**Performance benefit of patient-specific optimization.** (**A** and **B**) Average RS across number of electrodes for optimized LTL (**A**) and LH (**B**) configurations (*n* = 12). Shading shows standard deviation. (**C** and **D**) Reduction in RS for 11 transferred configurations compared with the patient-specific optimized configuration in each patient at a 500 µV threshold. The number of electrodes included in each configuration is the minimum number that yielded ≥75% RS in the optimized configuration. Configurations were optimized for LTL ROIs (**C**) and LH ROIs (**D**). The box plots describe median, maximum, minimum and interquartile range, with outside points indicating outliers.

Statistical analyses were conducted on MATLAB using built-in functions. The data are presented as median with interquartile range (Fig. 2), standard box plots (Figs 3 and 4C and D; Supplementary Fig. 2; median, interquartile range, maximum and minimum), mean ± SD (Fig. 4A and B) and standard histograms (Supplementary Fig. 5B and C) based on the characteristics of the data sets. A score of *P* < 0.05 was considered to be statistically significant. Shapiro–Wilk normality tests were conducted on each set of RS and electrode number data (Fig. 3). If the optimized and implanted data were both Gaussian for one threshold and one ROI, the difference between the two was compared using a two-tailed paired *t* test, and if not, using a Wilcoxon signed rank test (TL: *n* = 8; clinician-ROI: *n* = 12).

## Results

Clinical strategies for sEEG electrode implantation maximize the number of electrodes placed in an ROI that avoid vasculature and critical structures. This approach assumes that recordable signals are inherently localized and are generated by neural activity <5 mm away from a contact.^22^ However, the true spatial extent of RS is unknown. Optimization of sEEG electrode trajectories to maximize RS requires a robust understanding of how the complex source geometry and electrical conductivities of the brain influence the spatial extent of RS. Therefore, we first quantified the spatial extent of RS from simulated sEEG electrodes throughout the brain. Subsequently, we designed optimized sEEG electrode configurations that had substantially higher RS than clinically implanted configurations, and the optimized configurations required fewer implanted electrodes to achieve as good as or better RS than the clinically implanted configurations.

### sEEG contacts record discernible signals from realistic epileptiform neural activity up to 1.5 cm away

To determine the distance that sEEG contacts can record discernible signals from neural (epileptiform) sources, we developed a semi-automated patient-specific head modelling pipeline to simulate the voltages generated by cortical dipole sources throughout the brain (Fig. 1). We simulated epileptiform sources generating interictal spikes as patches of active cortex with 10 cm^2^ area^23^ and 0.465 nA-m/mm^2^ source strength at the peak of the spike.^16^ Sources with origins as far as 1.5 cm from the recording contacts generated readily detectable voltages (>500 µV at 20% sensitivity threshold; Fig. 2C), but patches of active cortex are spatially extended with an average radius of 1.39 cm for 10 cm^2^ patches (Supplementary Fig. 5). Therefore, intracranial electrode contacts generally record readily detectable signals from neural sources where a portion of the source is coincident with the recording electrode. However, there was large variability across contacts in RS (percent of sources that are recordable) [Eq. (1)] as a function of distance from the contact, and in some cases, larger signals were seen at farther distances from the contact compared with closer distances (Fig. 2A–C). Therefore, there is a complex relationship between contact location and RS that necessitates patient-specific extended source modelling to accurately determine the RS of each electrode contact.

There is uncertainty in the area and strength of the source used to represent epileptiform activity, and we quantified the recording sensitivity across a range of physiologically relevant source areas^23^ (6, 10 and 20 cm^2^) and strengths^16^ (0.16, 0.465 and 0.77 nA-m/mm^2^). We observed similar trends of RS as a function of distance across source areas and strengths but with substantial differences in scale where the strongest source (20 cm^2^ and 0.77 nA-m/mm^2^) generated signals >200 µV up to 3.5 cm away, while the weakest source (6 cm^2^ and 0.16 nA-m/mm^2^) never achieved >20% sensitivity for signals >200 µV (Fig. 2D).

### Optimized sEEG configurations yielded higher RS than clinically implanted configurations

Because sEEG contacts can record appreciable signals from neural sources located a few centimetres away from the recording site, there is a probable overlap in the regions recorded by multiple electrodes, and optimization can determine a set of sEEG trajectories with high RS. We optimized sEEG configurations to record from the LTL, LH and clinician-defined ROIs for 12 patients implanted with sEEG electrodes (Supplementary Table 1). The optimization algorithm iteratively identified electrode trajectories that minimized the number of regions (cortical patch sources) in the ROIs that were not yet recordable.

Optimized configurations exhibited far broader coverage of the ROI compared with clinically implanted configurations using the same number of electrodes (Fig. 3A and B). Optimized configurations had significantly higher recording sensitivities (2–47% higher) than implanted configurations in all patients for all voltage thresholds in both the LTL and the clinician-defined ROIs (*P* < 0.02; Fig. 3D and E). Further, optimized configurations required significantly fewer electrodes (0–11 fewer; *P* < 0.02) than implanted configurations to achieve the same or better RS (Fig. 3F and G). Finally, the RS of optimized configurations was higher than implanted configurations across all source areas, source strengths, signal detection thresholds and ROIs (Supplementary Fig. 2).

### Patient-specific optimization is necessary

Optimized electrode configurations were more sensitive than those implanted clinically, and we sought to determine whether patient-specific optimization was necessary or whether there were consistent optimal configurations to map the LTL and LH across patients. Patient-specific electrode configurations yielded comparable recording sensitivities as a function of the number of implanted electrodes across all subjects (Fig. 4A and B, Supplementary Table 2). However, the sets of optimized electrode trajectories were not consistent across patients (Supplementary Fig. 3). To quantify the benefit of patient-specific optimization, we transferred each optimized configuration to all other patients and compared the recording sensitivities of the patient-specific and transferred configurations. The transferred configurations had on average 30.5% lower RS than the patient-specific configurations for LTL ROIs (Fig. 4C) and 20.1% lower RS for LH ROIs (Fig. 4D). Further, all of the transferred configurations had invalid electrodes that intersected sulci and performance would decline further if these were removed. Therefore, standard optimal configurations did not exist, and patient-specific optimization was required to maximize the RS of sEEG electrode configurations.

## Discussion

### Clinical implications

Optimized sEEG electrode configurations, based on patient-specific models, yielded substantial increases in RS, and this may lead to a more accurate delineation of the EZ, and, thereby, improved outcomes of surgical resection. Further, the optimized configurations had RS equivalent to or greater with fewer electrodes than the clinically implanted configurations, and this may reduce the risk for complications, including intracranial haemorrhage.

We implemented and evaluated an optimization algorithm to determine implantation trajectories to map any clinician-defined ROI. Previous automated implantation trajectory planning algorithms were focused exclusively on finding ‘safe’ trajectories that intersected an ROI and did not incorporate measures of RS.^6-9^ Our optimized electrode trajectories produced larger cortical coverage than manual trajectory planning and thus had a greater probability of recording relevant epileptiform activity.

The RS of optimized configurations transferred across patients was inferior to the sensitivity of patient-specific configurations. Differences in the RS of individual contacts and differences in cortical geometry between patients influenced RS more than the general location of the electrodes. Therefore, standard configurations that perform well across most patients may not exist, and optimization of sEEG trajectory planning requires patient-specific modelling.

Our methods to calculate the RS of any electrode configuration enable clinicians to visualize the coverage generated by their preoperative plan in comparison with the ROI. They can then add, remove and move electrodes to create configurations with high RS within the ROI. These methods transform the clinical use of sEEG from a discrete *ad hoc* sampling to an intelligent, data-informed mapping of the ROI.

### Implications for source localization

The distances from an electrode contact that readily detect neural sources were similar to the spatial extents of neural sources (1.5 versus 1.39 cm for 10 cm^2^ and 500 µV threshold). Therefore, for large recorded signals, a portion of the neural source is likely to be <5 mm from the electrode contact. However, clinically, the size and centre of the source are used to design resection plans, and these source parameters cannot be intuitively determined because there is large variability in the RS of sEEG electrode contacts as a function of distance from the contact. Sources near the electrode contact may generate smaller signals compared with those further away, based on the orientation of the sources within the active tissue and the location of the contact. Therefore, delineating the centre of an epileptic source requires the use of sEEG source localization algorithms.^5,24-26^ Even though the RS for large amplitude signals is limited, source analysis with these signals is useful and accurate for sEEG.^5,26^ Additionally, our RS metric identifies the cortical regions that can be recorded by a set of sEEG electrodes, and this information can be used to constrain localization of the EZ, which will simplify the source localization problem and thereby improve accuracy.

Neural sources >200 µV were distinguishable from noise, while most epileptiform signals are analysed only if they are >1000 µV. If only large signals are localized clinically, epileptic sources that generate smaller signals and are slightly farther from the electrodes may be missed. Recent spatiotemporal source localization algorithms using EEG reconstructed neural signals having a signal-to-noise ratio as low as 5 dB.^27^ Therefore, new source localization algorithms might be able to take advantage of lower-amplitude signals (200 µV) to localize neural sources that were previously not visible to the clinician. Further, detecting lower-amplitude signals allows greater RS and fewer electrodes to be used in optimized electrode configurations.

### Modelling limitations

Quantification and optimization of RS using patient-specific models were computationally intensive. The full simulation and analysis for each patient took an average of 22 h on up to 400 CPUs on the Duke Computing Cluster, equivalent to ∼278 days of gross compute time on a single CPU. Also, our comparisons may be limited by the manually defined ROIs. Our algorithm is agnostic to the preferences of ROI subregions that clinicians may have based on specific hypotheses of EZ location. To address this limitation, our algorithm could be used in a two-tiered search process to map small regions (e.g. hippocampus) with high thresholds and then larger regions (e.g. TL) with lower thresholds. Alternately, our algorithm could be used in conjunction with manual trajectory selection. Given some number of clinician-defined electrode locations, we can calculate and exclude the initial recordable area from the user-defined ROI. Then, our methods can find optimal electrode locations to record from the remaining area.

Our work demonstrates clear performance benefits of optimization of electrode configurations, and future implementations could also include more accurate methods to avoid critical anatomy (vasculature, ventricles). Our methods to generate sulcal surfaces selected large areas in the TLs and likely overestimated the area around large blood vessels and thereby limited the search space for valid electrodes in the TLs. While sulcal surfaces have been used for vasculature avoidance,^7^ vasculature surfaces can be extracted using CT angiography. Direct avoidance of vasculature could improve the safety profiles of trajectories and increase the search space of valid electrode implantation areas. Additionally, while all optimized configurations satisfied conservative risk metrics, future optimization methods could not only maximize RS but also minimize the risk of configurations.^7,8^

Another limitation is the accuracy of our epileptic source models. While dipoles are appropriate source models for sEEG, there are errors in voltages generated by dipole models compared with realistic neurons within 1 mm of sources,^15^ where higher-order components of the current multipole dominate the peak of voltage spikes.^28^ Because our patch dipole models extend millimetres from the central dipole, we may be underestimating the amplitude of signals within 5 mm of patch centres (Fig. 2). Furthermore, there is uncertainty in the dipole moment density and patch area parameters, and our approximations are based on interictal spike signals only and may not represent well other epileptiform signals (i.e. high frequency oscillations and ictal spikes). Because most contacts could not record any signals >500 µV at any distance for 6 cm^2^ sources (Fig. 2D) and 500 µV signals are commonly recorded in sEEG (Supplementary Fig. 1), epileptic sources may be larger and/or stronger than 6 cm^2^ and 0.16 nA-m/mm^2^, respectively. Our estimates of recording radius and RS are dependent on these parameters, and further refinements of these estimates would be beneficial. However, regardless of the specific source parameters used, we quantified RS and identified electrode configurations for any ROI with higher RS than clinically implanted configurations. Finally, proper optimization of electrode trajectories is dependent on knowing the source parameters of the true neural source, which are currently unknown, and errors can arise from optimizing electrode trajectories with one set of source parameters that are different from the true neural source (Supplementary Fig. 4).

## Supplementary Material

fcad304_Supplementary_Data

## Data Availability

The code and data that support the findings of this study are openly available in the GitLab repository at https://gitlab.oit.duke.edu/ged12/seeg-rec-sensitivity and https://doi.org/10.5281/zenodo.7604030.
