# Supplementary material for: Optimization of patient-specific stereo-EEG recording sensitivity
Source: Brain Commun. 2023 Nov 2;5(6):fcad304. doi: 10.1093/braincomms/fcad304 (PMC10655844; doi:10.1093/braincomms/fcad304)
Supplement: fcad304_Supplementary_Data [file fcad304_supplementary_data.pdf]

Optimization of Patient-Specific Stereo-EEG Recording Sensitivity

Supplementary Material

Supplementary Methods

We performed a sensitivity analysis to assess the robustness of our optimization methods to uncertainty in source parameters. Since the true source parameters of epileptic sources are unknown, we sought to determine the recording sensitivity error associated with using the wrong source type. We computed optimized configurations for all nine source types, 12 patients, and the LTL and LH ROIs. We analyzed the recording sensitivity for each configuration with all nine source types (one matched case and eight “crossed” cases). We quantified the error due to source uncertainty by calculating the percent error in recording sensitivity between the crossed and matched cases for every source type. We used the minimum number of electrodes in each configuration such that the matched recording sensitivity was ≥75%. We used a 500 μV-priority cost function in all optimization cases, a 500 μV threshold for analysis, and averaged the percent error across all 12 patients.

We also performed a sensitivity analysis to quantify the dependence of our optimization results on threshold-priority ordering of the cost function. Using the mean source type, we found optimized configurations for all six permutations of threshold-priority ordering of the cost functions, all 12 patients, and the LTL and LH ROIs. We compared the recording sensitivity for each configuration with all three thresholds (200, 500, 1000 μV), giving us two matched cases (first priority threshold matches analysis threshold) and four crossed cases (first priority threshold does not match analysis threshold). We used the minimum number of electrodes in each configuration such that the maximum recording sensitivity across cases was ≥75%, and we quantified the percent error in recording sensitivity between the best case and all other threshold-priority cases. We averaged the error across all 12 patients.

We quantified the projected area and radii of our source models to compare more directly to recordable radius (Supplementary Fig. 5). For a single patient, we calculated the area and mean radius of our sources projected onto the inside of the skull for all patches of three source areas (6 cm^2, 10 cm^2, and 20 cm^2). We excluded all patches whose maximum distance to the skull was >3 cm because patches far from the skull (i.e., in the midline and the insula) did not have a clear projected area (18832 of 39995 sources). For the remaining sources, we found the projected patch by first selecting the closest point on the skull surface to each of the vertices of the cortex patch. To smooth the boundary and fill in holes, we dilated and eroded the patch vertices three times each. We then selected the faces whose vertices were all included in the set and took the corresponding area. We calculated the mean radius between the centroid and all boundary points in the projected patch.

We conducted a two-factor ANOVA to analyze the recording radius across cortical subregions and patients. We calculated the maximum recording radius (largest binned radius with ≥20% recording sensitivity) for each of the 105 patches for each patient using the median source type (10 cm^2 area and 0.465 nAm/mm^2 dipole moment density) and all three voltage thresholds (200 µV, 500 µV, and 1000 µV). We grouped the patches based on the subregion of their central face according to the Desikan-Killiany atlas and used paired t-tests with Bonferroni corrections for post hoc testing.

Supplementary Results

Assuming that the mean source type was ground truth, we computed the error in optimizing with all other source types. We had a maximum average error of 24% for the LTL (Supplementary Fig. 4AC, boxed row) and 27% for the LH (Supplementary Fig. 4BD, boxed row). Assuming that optimization was run using the mean source type, we also quantified the error associated with

the ground truth source type being any of the other source types. We had a maximum average error of 38% for the LTL (Supplementary Fig. 4C, boxed column) and 58% for the LH (Supplementary Fig. 4C, boxed column). However, the difference in dipole moment density dominated the errors, and the mean source type optimizations (boxed columns) had ≤17% error excluding 0.16 nAm/mm^2 cases.

The error in recording sensitivity between threshold-priority order cases with the same first priority threshold was small (≤3.3%) (Supplementary Fig. 4E-H). Using 500 µV-priority order in optimization, there was ≤17% error in recording sensitivity for the LTL and ≤25% for the LH when compared to optimizations with different first priority thresholds (Supplementary Fig. 4G-G). The only large error was for 200 µV-priority thresholds analyzed at 1000 µV for LH configurations (41%) (Supplementary Fig. 4H). Therefore, the choice of first priority threshold is very important for appropriate optimization.

For the projected patch analysis, we found that the 10 cm^2 sources had an average projected area of 3.85 cm^2 and mean radius of 1.39 cm (Supplementary Fig. 5). The average projected area was ~40% the surface area of source models (6 cm^2, 10cm^2, 20cm^2).

For the 200 µV voltage threshold, we found a significant difference in the maximum recording radius across cortex subregions (2-factor ANOVA; $P < 0.001$, F = 2.15). For the other thresholds (500 µV and 1000 µV), we found no significant difference in the maximum recording radius across cortex subregions (2-factor ANOVA; $P = 0.093$, F = 1.34). For all three thresholds, we found a significant difference in the maximum recording radius across patients ($P < 0.0001$). We included test statistics for all tests in Supplementary Table 4A.

Although we found a significant difference in maximum recordable radius for the 200 µV threshold across cortex subregions, post hoc testing revealed no significant differences between any pairs of temporal subregions (transverse temporal, superior temporal, middle temporal, inferior temporal, fusiform, temporal pole, para-hippocampal, and entorhinal cortex) (Supplementary Table 4B-C). All significant pairs (4 of 595) included the inferior parietal region.

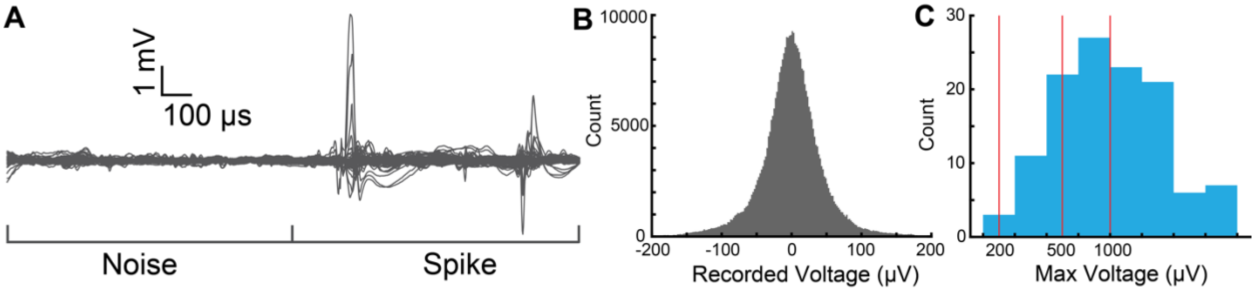

Supplementary Figure 1: Estimation of discernible recording threshold. A Example signals of sEEG background noise and an interictal spike. **B** Distribution of noise amplitude. **C** Distribution of interictal spike amplitude (log scale).

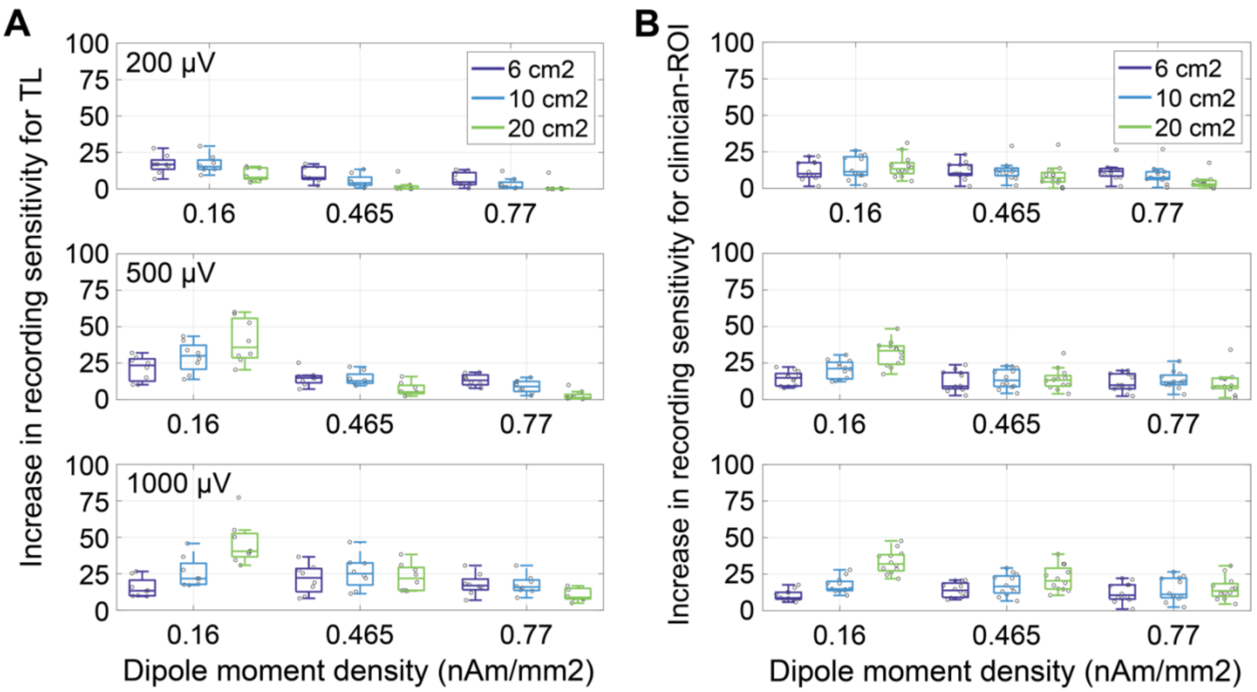

Supplementary Figure 2: Optimized configurations have increased recording sensitivity. Increase in recording sensitivity between optimized configurations and clinically implanted configurations across all dipole moment densities and patch areas at three signal detection thresholds. **A** Configurations for temporal lobe ROIs (n = 8). **B** Configurations for full clinician-defined ROIs (n = 12). Both figures show standard box plots with median, interquartile range, maximum and minimum.

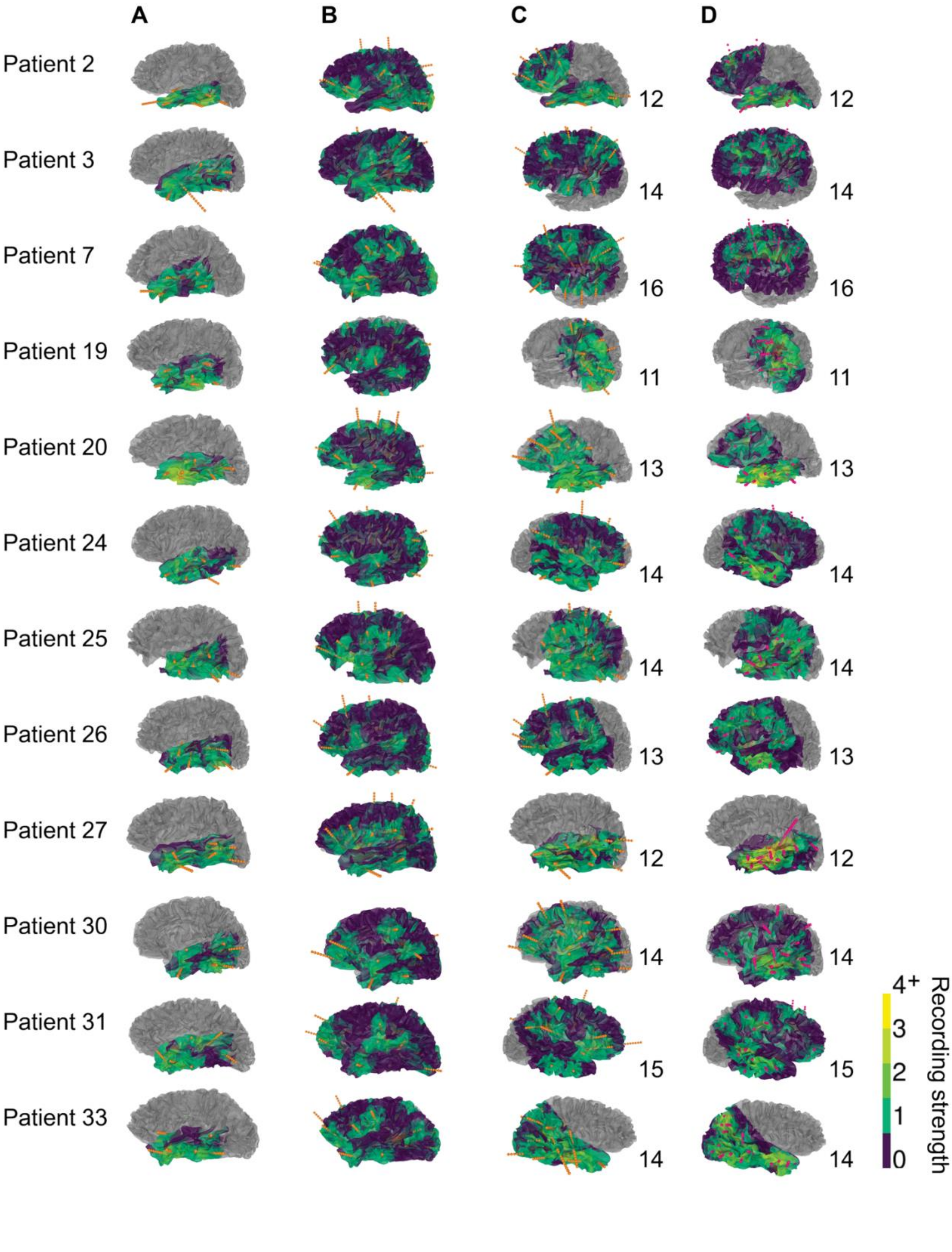

A
B
C
D
Patient 2
12
12
Patient 3
14
14
Patient 7
16
16
Patient 19
11
11
Patient 20
13
13
Patient 24
14
14
Patient 25
14
14
Patient 26
13
13
Patient 27
12
12
Patient 30
14
14
Patient 31
15
15
Patient 33
14
14
4+
3
2
1
0
Recording strength

Supplementary Figure 3: Optimized and clinically implanted configurations for 12 patients and three regions of interest (ROIs). Colored spheres represent electrode contacts, colored cortex indicates recording strength in the ROI, and grey area is outside the ROI. Configurations were not able to record from the purple cortex**. A** Optimized configurations for LTL with six electrodes**. B** Optimized configurations for LH with 12 electrodes**. C-D** Optimized **(C)** and implanted **(D)** configurations for the clinician-defined ROI with the number of electrodes noted.

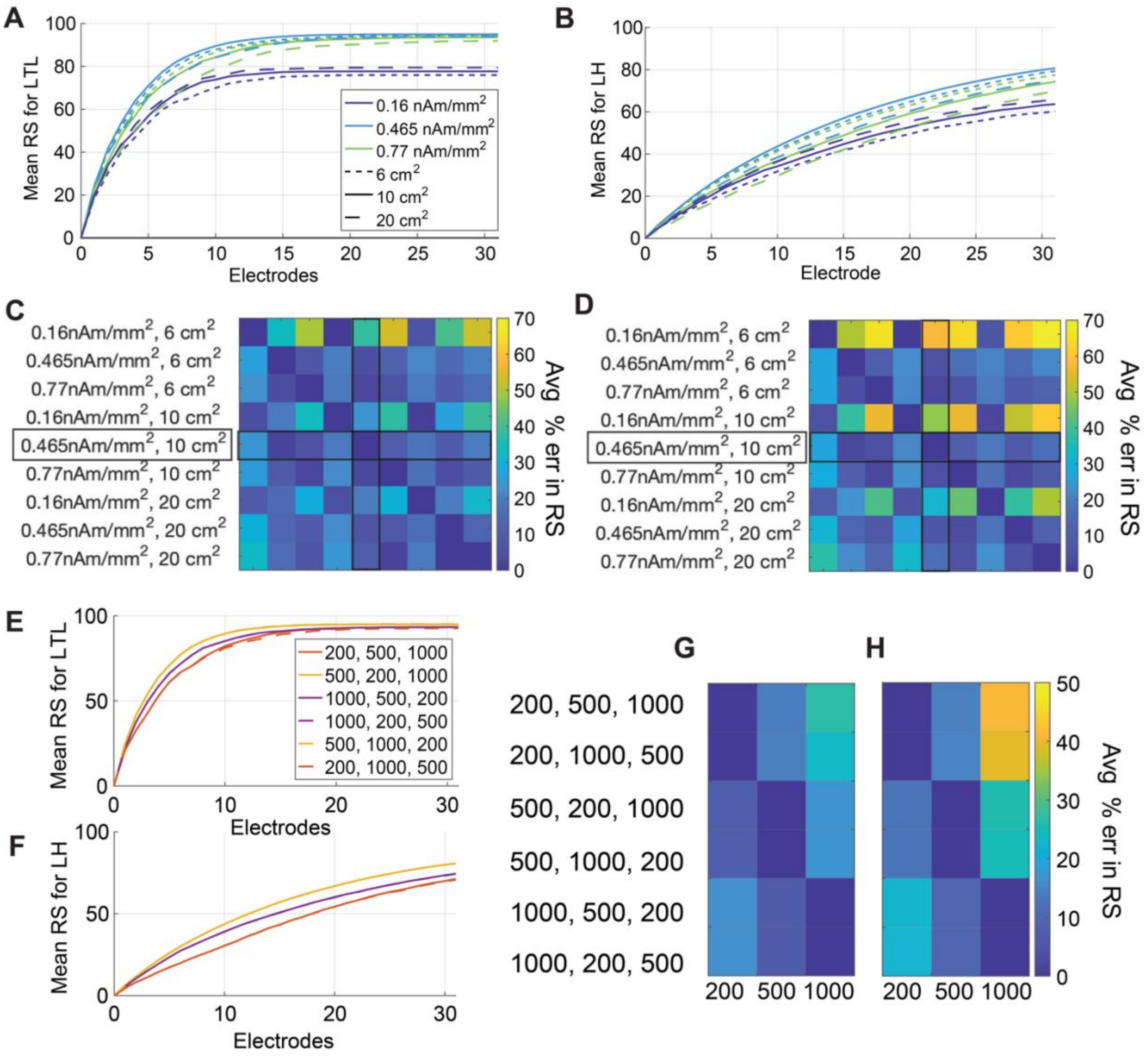

Supplementary Figure 4: Sensitivity of optimized configuration recording sensitivity to source model parameters and threshold priority order. A-B Recording sensitivity (RS) of LTL configurations (**A**) and LH configurations (**B**) averaged across 12 patients as a function of number of electrodes, using the mean source type (10 cm^2 and 0.465 nAm/mm^2) for RS calculation and all nine source types as optimization parameters. The top series (0.465 nAm/mm^2, 10 cm^2, solid blue line) corresponds to the matched case of both optimization and RS calculation with the mean source type. **C-D** Percent error in RS due to uncertainty of source

modeling parameters for all nine source types, for LTL configurations (**C**) and LH configurations (**D**), averaged across all 12 patients. Rows represent source parameters of analysis, and columns represent source parameters of optimization. All data in **A-D** correspond to a 500 µV discernible voltage threshold and a 500 µV-priority cost function. **E-F** Recording sensitivity (RS) for LTL (**E**) and LH (**F**) configurations averaged across all 12 patients as a function of number of electrodes, for all six possible threshold-priority orders, analyzed at 500 µV. **G-H** Average percent error in RS for 12 patients in LTL (**G**) and LH (**H**) configurations for six threshold-priority orders in configuration optimization (rows) and three thresholds for analysis (columns).

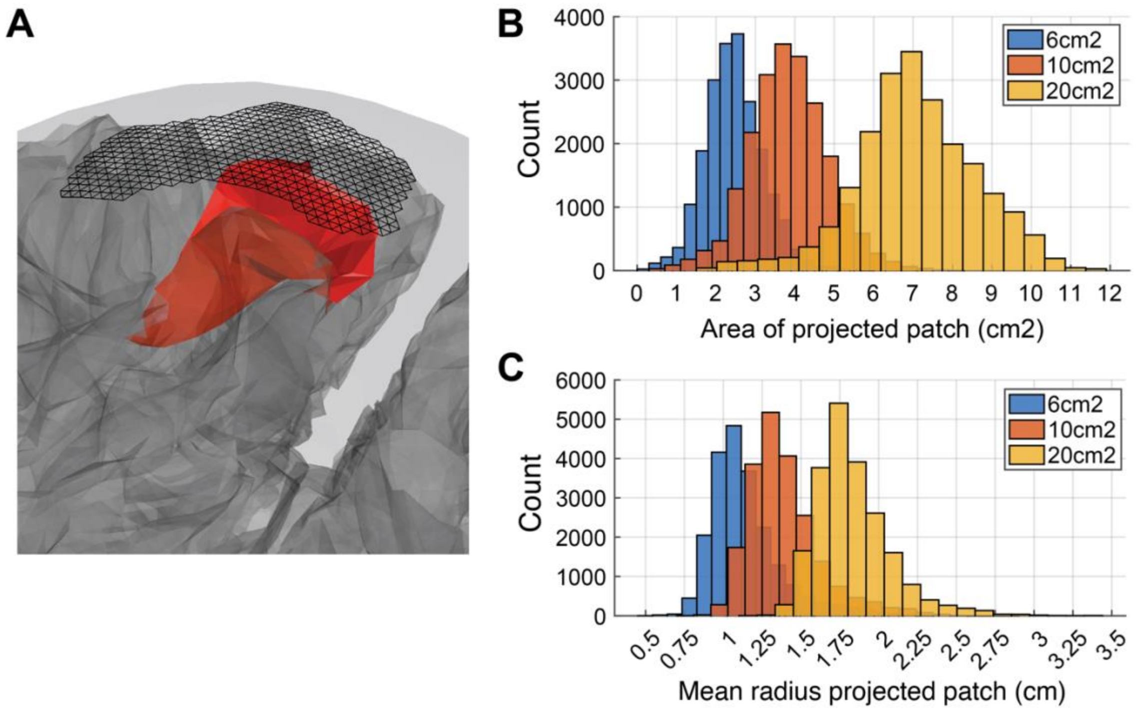

Supplementary Figure 5: Area and mean radius of sources projected onto the inside of the skull. A 10.00 cm^2 patch (red) on the cortex surface (dark gray) with projected patch (black) on the inner skull surface (light gray) for patient 25. The projected patch has an area of 4.94 cm^2 and a mean radius of 1.30 cm. **B** Histograms of projected patch area for 21,163 patches across three cortex patch sizes. **C** Histograms of mean radius of projected patch for 21,163 patches across three cortex patch sizes. The average projected patch areas are 2.46, 3.85, and 7.09 cm^2 and the average mean radii are 1.14, 1.39, and 1.83 cm for 6, 10, and 20 cm^2 patches, respectively.

Supplementary Table 1: SEEG patient population specifications

Patient ID	Gender	Implantation Hypothesis	Total # Electrodes	Included for TL hypothesis (# Electrodes)
2	M	L TEMPORAL/INSULA; L TEMPORO-OCCIPITAL LESION	12	Yes (6)
3	F	L>R FRONTO-PARIETAL, NEAR MIDLE	14	No
7	F	FRONTAL>PARIETAL, UNCLEAR LATERALIZATION	16	No
19	M	L OCCIPITAL PERILESIONAL	11	No
20	F	L LIKELY T, BUT NOT MESIAL; CONCERN FOR TEMPORAL PLUS	13	Yes (9)
24	F	R HEMISPHERE, LIKELY T; BUT NEED COVERAGE OF CINGULATE, INSULA, ORBITOFRONTAL; L MESIAL T COVERAGE AS WELL	14	Yes (6)
25	F	LEFT>RIGHT TEMPORO-PARIETAL, INCLUDING CINGULATE/POSTERIOR INSULA COVERAGE; SOME R TEMPORAL PLUS COVERAGE AS WELL	18	Yes (10)
26	M	L>R, FRONTAL>TEMPORA; BUT CONCERN FOR MULTIFOCAL	16	Yes (4)
27	F	L TEMPORAL, LIKELY LATERAL	12	Yes (10)

30	F	L, SUPSECT TEMPORAL>FRONTAL, LATERAL>MESIAL (ESPECIALLY SUPERIOR T GYRUS)	14	Yes (8)
31	F	R TEMPORO-PARIETAL REGION; NEED COVERAGE OF R T+, INCLUDING POSTERIOR T/SUPERIOR T GYRUS, CINGULAGE, ORBITOFRONTAL	15	Yes (6)
33	F	R OCCIPITAL, POSTERIOR TEMPORAL>PARIETAL	14	no

Supplementary Table 2: Number of electrodes for ≥75% mean recording sensitivity for standard optimized configurations with standard deviation.

	LTL	LH
200 µV	3 +/- 0.7	11 +/- 1.9
500 µV	6 +/- 1.4	26 +/- 3.6
1000 µV	12 +/- 3.9	> 30

Supplementary Table 3A: Test statistics for Figure 3D-E. Recording sensitivity.

ROI	Threshold (µV)	Test used	P value	Test statistic (t value or z value)
TL (Figure 3D)	200	Wilcoxon signed rank test	0.0117186856	2.520504151
	500	ttest	0.0000533723967	8.694443568
	1000	ttest	0.000374733762	6.378913292
Clinician-ROI (Figure 3D)	200	ttest	0.000045547084	6.479360262
	500	ttest	0.0000138055366	7.388000363
	1000	ttest	0.00000422862486	8.372111984

Supplementary Table 3B: Test statistics for Figure 3F-G. Number of electrodes.

ROI	Threshold (µV)	Test used	P value	Test statistic (t value or z value)
TL (Figure 3F)	200	ttest	0.00582040544	-3.910404344
	500	ttest	0.000391428146	-6.333333333
	1000	Wilcoxon signed rank test	0.0114120364	-2.529822128
Clinician-ROI (Figure 3G)	200	ttest	0.00000264209782	-8.789038116
	500	ttest	0.0000172292594	-7.213329322
	1000	ttest	0.000000720601308	-10.02566253

Supplementary Table 4A: Two-factor ANOVA statistics for maximum recording radius across cortex subregion and patients for the median source type on all three discernible voltage thresholds. Bold values show significance ($P < 0.05$).

Threshold	Variable	F value	*P* value
200 µV	subregion	2.15455552450142	**0.000150873884616753**
	patient	15.3382856857261	**2.63988780485527e-28**
500 µV	subregion	1.34001887914335	0.0929653886755033
	patient	5.89887291281083	**2.10964634192425e-09**
1000 µV	subregion	1.01760469187042	0.440915689128699
	patient	4.15541964046545	**4.78242441910914e-06**

Supplementary Table 4B: T-values matrix for post hoc t-tests for maximum recording radius between temporal regions for all patients with a 200 µV threshold. No pairs are significant. Lateral temporal regions are shaded gray.

	Inferior temporal	Middle temporal	Superior temporal	Temporal pole	Transverse temporal	Para hippocampal	Entorhinal	Fusiform
Inferior temporal		-0.83333	-0.75000	-0.72222	-0.44444	0.11111	0.83333	0.63889
Middle temporal	-0.83333		0.083330	0.11111	0.38889	0.94444	0	-0.19444
Superior temporal	-0.75000	0.083330		0.027780	0.30556	-0.86111	0.083330	-0.11111
Temporal pole	-0.72222	0.11111	0.027780		0.27778	-0.83333	0.11111	-0.08333
Transverse temporal	-0.44444	0.38889	0.30556	0.27778		-0.55556	0.38889	0.19444
Para hippocampal	0.11111	0.94444	-0.86111	-0.83333	-0.55556		0.94444	0.75000
Entorhinal	0.83333	0	0.083330	0.11111	0.38889	0.94444		0.19444
Fusiform	0.63889	-0.19444	-0.11111	-0.08333	0.19444	0.75000	0.19444	

Supplementary Table 4C: P-value matrix for post hoc t-tests for maximum recording radius between temporal regions for all patients with a 200 µV threshold. No pairs are significant.

	Inferior temporal	Middle temporal	Superior temporal	Temporal pole	Transverse temporal	Para hippocampal	Entorhinal	Fusiform
Inferior temporal		1	1	1	1	1	1	1
Middle temporal	1		1	1	1	1	1	1
Superior temporal	1	1		1	1	1	1	1
Temporal pole	1	1	1		1	1	1	1
Transverse temporal	1	1	1	1		1	1	1
Para hippocampal	1	1	1	1	1		1	1
Entorhinal	1	1	1	1	1	1		1
Fusiform	1	1	1	1	1	1	1	
